# Thermal Stress Analysis and Control Method for Surface Acoustic Wave Atomizer

**DOI:** 10.3390/s23218748

**Published:** 2023-10-26

**Authors:** Xufeng Xue, Baile Cui, Xianping Chen, Wen Wang, Mingchen Sun, Yong Liang

**Affiliations:** 1 College of Optoelectronic Engineering, Chongqing University, Chongqing 400044, China; xuexufeng@mail.ioa.ac.cn (X.X.); xianpingchen@cqu.edu.cn (X.C.); 2 Institute of Acoustics, Chinese Academy of Sciences, Beijing 100190, China; cuibaile@mail.ioa.ac.cn (B.C.); sunmingchen@mail.ioa.ac.cn (M.S.); liangyong@mail.ioa.ac.cn (Y.L.); 3 School of Electronic, Electrical and Communication Engineering, University of Chinese Academy of Sciences, Beijing 100049, China

**Keywords:** atomization, thermal stress, surface acoustic wave

## Abstract

To prevent the potential failure of the surface acoustic wave (SAW) atomizer caused by the concentration of thermal stresses, this study investigates the thermal elevation process inherent to the operation of the surface wave atomizer. Subsequently, a method for temperature regulation is proposed. By collecting the temperature rise data of SAW atomizers with water, olive oil, and glycerol at 5/6/7 Watts (W) of power, the temperature curves of the atomizer surface under different conditions are obtained, and the stress changes in the working process are simulated additionally. The results indicate that although the stress generated by surface acoustic wave atomizers varies for different media, there is always a problem of rapid heating during the initial working stage in all cases. To address the above issues, this study analyzed the time when the maximum stress occurred and proposed control methods based on experimental data. The simulation results show that by controlling the driving power within 4 s after the start of atomization, the problem of excessive stress during the heating stage can be avoided. Finally, the feasibility of the control method was verified through a simple power control method (limiting the driving power to 3 W in the first 2 s), proving that this method can effectively reduce the thermal stress during the working process of the atomizer and prevent the atomizer from cracking.

## 1. Introduction

The surface acoustic wave atomizer makes use of the energy of the surface acoustic wave (SAW) to achieve atomization, which has the advantages of high efficiency, low energy consumption, controllable atomization particle size, fast response, etc. It can be widely used in a great number of fields, including medicine [1], tobacco [2], gene chip [3], chemistry [4,5,6], and so on.

The principle governing the surface acoustic wave atomizer primarily involves the propagation of high frequency acoustic waves into a liquid medium. The energy carried by these acoustic waves leads to the deformation of the liquid, resulting in the formation and ejection of minute liquid droplets. Specifically, SAW is typically generated by interdigital transducers on the surface of a piezoelectric crystal. Due to the piezoelectric effect of the crystal, the interdigital transducers produce mechanical vibration when a high frequency alternating voltage is applied, generating SAW on the piezoelectric crystal surface. As surface acoustic waves propagate along the surface of a piezoelectric crystal, the application of a liquid coating onto the crystal’s surface allows these waves to penetrate the liquid medium. Due to the vibrational characteristics of SAW, it will cause tiny vibrations on the liquid surface. These vibrations lead to the formation of small liquid droplets, which continuously form, disperse, and rupture as SAW propagates, resulting in the generation of atomized mist.

During the atomization process, when molecules or particles in the liquid are exposed to high-frequency vibrations, they experience mutual friction, which converts kinetic energy into heat, causing the liquid to heat up [7,8]. In medical, tobacco, chemical, and other fields, common applications require a large amount of aerosol. A more powerful drive for the atomizer must be employed to achieve a larger atomization output. However, a higher power level can lead to stronger thermal effects. In the fields of health, beauty, and tobacco, where plant essential oils and glycerol are the main atomizing objects, this thermal effect is particularly severe. Plant essential oils and glycerol have high boiling points and high viscosity, leading to an extremely fast heating rate of the atomizer. Their working temperature generally exceeds 100 °C. In experimental tests, it was found that under high power conditions, their working temperature can even reach 200 °C. The sharp temperature gradient caused by the rapid temperature rise of the atomizer results in different thermal expansions in different parts of the material. If the thermal stress exceeds the material’s strength threshold, it precipitates the initiation and propagation of cracks. These cracks will damage the integrity of the device, reduce its performance, and eventually lead to device failure. In the study conducted by Qing-Yun Huang et al., the maximum temperature difference measured on the atomizer surface during atomization reached 76.2 °C, which is the main cause of device rupture [9].

To address device failure caused by thermal stress, current research typically focuses on enhancing heat dissipation design [9] and reducing driving power [10]. In the study by Qing Yun Huang et al., the temperature difference on the atomizer surface was reduced from 76.2 °C to 15 °C by using silver slurry with better thermal conductivity as the adhesive [9]. In the study by Han, J. et al., it was proposed that device damage can be avoided by enhancing the heat dissipation efficiency by using adhesives with higher thermal conductivity, enhancing the heat dissipation structure design, and increasing surface thermal convection, such as using fans [11]. In the study by Chenhui Gai et al., a method was proposed to regulate the thermal distribution of devices by adjusting the duty cycle of the driving signal and changing the hydrophilicity of the substrate [12].

Based on the above research results, the advantage of enhanced heat dissipation design is that it does not require adjusting the system drive level and does not affect the atomization amount. However, adhesives with high thermal conductivity, such as silver paste, can bring cost improvements, while the use of heat dissipation fins and fans can lead to an increase in the volume of the atomizer, which is limited in small-scale applications. The method of adjusting the duty cycle of the driving signal reduces the non-uniformity of the temperature distribution in the atomizer by reducing heat generation, but it will result in a decrease in atomization volume.

**Figure 1:**
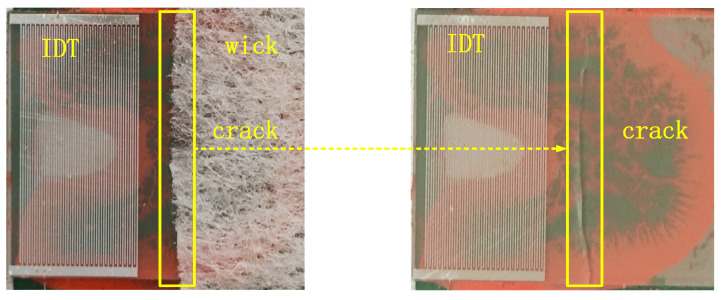
Atomizer rupture failure.

Although the current research has proposed the above methods to solve the problem of thermal stress concentration, there is no detailed analysis of when the maximum thermal stress will occur and how to control the heating rate of the atomizer surface. This is due to the lack of research on the stress change process during the working process of the atomizer. Understanding the stress changes of atomizers under high-temperature operating conditions is particularly important for the application of a high boiling point, high viscosity, and high mist-content liquid media. As shown in Figure 1, a typical atomizer fracture failure diagram caused by thermal effects is shown, with the fracture located at the edge of the liquid guide wick. The left image shows the complete atomizer that has undergone fragmentation, with the fragmentation position obstructed by the liquid guide wick. The right image shows the atomizer with the liquid guide wick removed, and the fragmentation marks can be clearly seen.

To address the aforementioned issues, this study aims to investigate stress variations in a SAW atomizer during the atomization process. By collecting temperature data of the atomizer under different media and power conditions, surface temperature curves of the atomizer under different scenarios are obtained. Simulation calculations are conducted to explore stress distribution during the operation of the atomizer and investigate the occurrence time and intensity of stress peaks. Based on these findings, effective methods to reduce thermal stress are proposed.

## 2. Measurement of Atomizer Thermal Distribution

**Figure 2:**
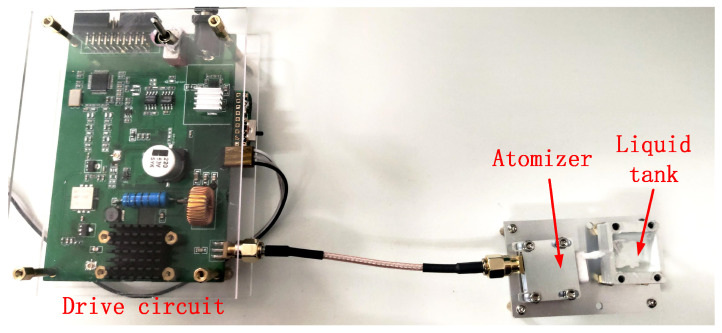
SAW atomizer test system.

This study employs an HM-TPH16-6VF/W infrared thermographic camera to record the temperature variation of the atomizer’s surface. This method provides contactless temperature measurement and real-time temperature distribution of the entire device surface. The test system used is shown in Figure 2, which includes a driver circuit located on the left and an atomizer and liquid tank located on the right. The thermographic camera takes photos directly above the atomizer.

The designed surface acoustic wave atomizer structure uses electron beam evaporation technology to deposit 800 nm metal aluminum electrodes on a smooth and flat 128° YX LiNbO${}_{3}$ substrate surface, with the device operating frequency set at 20 MHz. The interdigital transducer adopts a uniform IDT structure, with an interdigital (IDT) width and interdigital distance of 1/4 wavelength, and an aperture set to 50 wavelengths, which can excite surface acoustic waves in both the left and right directions. The blank area of the substrate on the right side of the interdigital transducer is the atomization area, and the surface of the atomization area is a capillary structure liquid wick that can adsorb liquid molecules (Figure 1). The atomizer developed is bonded to the surface of the heat-dissipation metal aluminum plate through a thermal conductive adhesive.

**Figure 3:**
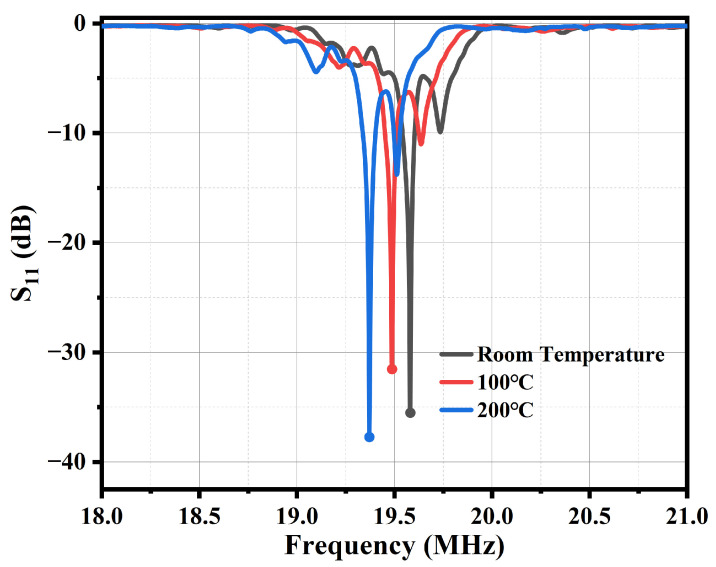
Frequency response of atomizer in different temperature.

The measurement of the return loss of the atomizer using the network analyzer E5061B is shown by the red curve in Figure 3. From the figure, it can be seen that the resonance frequency of the atomizer is about 19.5806 MHz, and the return loss is −35.516 dB. Considering that the significant temperature variation of the atomizer during the atomization experiment can lead to a shift in the resonance frequency of the atomizer, the atomizer was heated using a heating table and the resonance frequency of the atomizer under high temperature conditions was measured. The measurement results are shown by the black and blue curves in Figure 3. When the heating table is set at 100 °C, the surface temperature of the atomizer is measured to be 96 °C, its resonant frequency is about 19.4869 MHz, and the return loss is −31.5431 dB. When the heating table is set at 200 °C, the surface temperature of the atomizer is measured to be 182 °C, its resonant frequency is about 19.3706 MHz, and the return loss is −37.7357 dB. The decrease in device frequency is due to the frequency temperature coefficient of 128° YX LiNbO${}_{3}$ being −75 ppm/K [13], while the change in return loss is due to the influence of heating on material parameters and impedance matching.

From Figure 3, it can be seen that as the temperature increases, the resonant frequency of the atomizer gradually decreases. If the atomizer operates at around 100 °C, its driving frequency needs to be reduced by 0.1 MHz compared to the room temperature. If it operates at around 200 °C, its driving frequency needs to be reduced by about 0.2 MHz compared to the room temperature. In actual testing, due to the fast heating speed of the atomizer, it can reach a stable operating temperature within seconds. Therefore, the heating process can be ignored and the resonant frequency under stable operating conditions can be directly used for driving. For the atomizer shown in Figure 3, due to its stable working temperature for water atomization being close to 100 °C, a 19.5 MHz signal is used for driving. For glycerol or olive oil atomization, the stable working temperature is close to 200 °C, so a 19.4 MHz signal is used for driving.

Experiments were conducted to test the atomization heating using three kinds of liquid: water, olive oil, and glycerol. Each kind was tested with input power of 5 W, 6 W, and 7 W for comparison. To improve the accuracy of the experiments, each test was repeated three times, and all data were taken into account.

**Figure 4:**
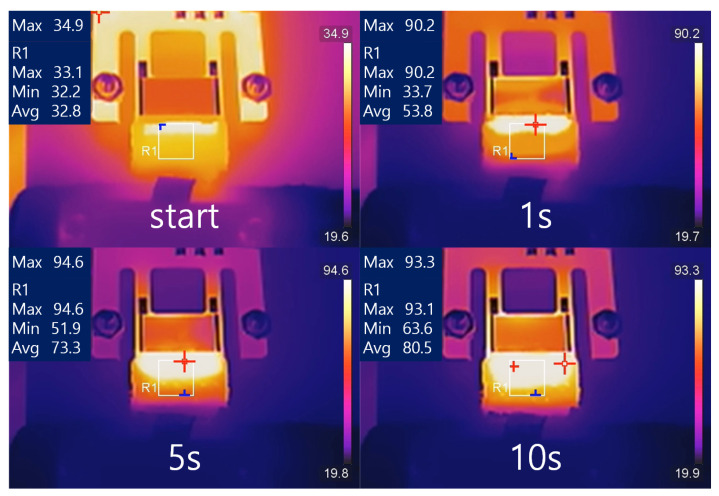
Temperature Distribution at Different Times: the red symbol (the highest point); the blue symbol (the lowest point).

Figure 4 shows the typical heating process during atomizer operation. In the figure, the numbers displayed in the upper left corner indicate the highest temperature in the entire region, the highest value in the R1 region, the lowest value in the R1 region, and the average value in the R1 region from top to bottom, in degrees Celsius. In the initial stage, the temperature of the atomizer surface rises rapidly, with the fastest temperature rise occurring at the front end of the wick where it first encounters SAW. After a few seconds of heating, the highest temperature on the atomizer surface reaches a stable state, and heat gradually spreads to other parts of the atomizer through thermal conduction. The example data in the figure represent the process of water heating with a 7 W input power. In the following parts, we will analyze different indicators such as the heating rate and maximum temperature under different experimental conditions in detail.

**Figure 5:**
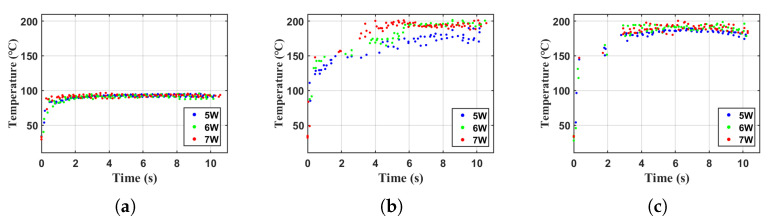
Different liquids temperature variation in 5/6/7 W power levels. (**a**) Water, (**b**) olive oil, (**c**) glycerol.

The three kinds of liquid were driven by signals with powers of 5 W, 6 W, and 7 W, and the temperature rise within 10 s was recorded as shown in the graph. Each point in the graph represents the highest temperature in the entire area captured by the infrared thermographic camera at the current time. From the data in Figure 5, it can be observed that in the initial stage, all the liquid undergoes rapid heating, reaching a stable state and maintaining a constant working temperature in a short time.

The working temperature in the stable state is mainly related to the boiling point and the overall heat dissipation efficiency. For water, its heating limit is the boiling point, 100 °C, and the measured stable working temperature is around 90 °C. For olive oil and glycerol, both of their boiling points are higher than 200 °C, and the measured stable working temperature is around 190 °C, which is not close to the boiling points and is related to the overall heat dissipation efficiency of the system. The initial rate of temperature rise is mainly related to viscosity. The viscosity of water is the lowest, about 0.9 CP at 20 °C. The measured heating results are shown in Figure 5a, with each point representing the highest temperature data at the current time. From the figure, it can be seen that the heating rate slightly increases with the increase in power. However, due to the faster heating and lower stable temperature, the difference is small. The viscosity of olive oil is slightly higher, about 80 CP at 20 °C [14]. The measured heating results are shown in Figure 5b, and its heating rate significantly accelerates with increasing power. The viscosity of glycerol is the highest, about 1412 CP at 20 °C [15]. The measured temperature rise results are shown in Figure 5c. As the driving power increases, there is no significant difference in the heating rate. This is because the excessively high heating rate makes it difficult to observe subtle differences.

## 3. Analysis of Thermal Stress

### 3.1. Temperature Variation Fitting Curves

**Figure 6:**
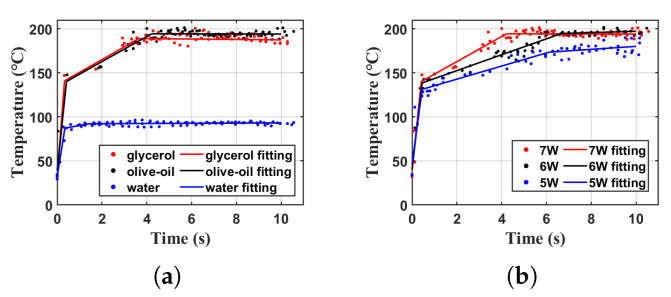
Temperature variation fitting curves. (**a**) Different liquids at 7 W power, (**b**) olive oil at different power Levels.

During the experiment, we observed that at the power level of 7 W, the atomization of water did not cause any damage to the atomizer structure. This phenomenon has been verified through many tests over several weeks. However, atomization experiments on glycerol and olive oil at the same power level of 7 W can quickly lead to internal structural damage. Therefore, stress analysis for the atomizer will be based on the data obtained at 7 W power conditions, and the stress analysis results for water atomization at 7 W will be used as the stress control. The fitting results for different liquids at 7 W Power are shown in Figure 6a. For the comparative analysis of the same liquid under different power conditions, the temperature data of water and glycerol varied only slightly under three power conditions, making the analysis results easily affected by errors during the measurement and fitting process. Therefore, the data for olive oil with a larger temperature difference were used for analysis, and the fitting results are shown in Figure 6b. The parameters of the fitting curves in Figure 6 are recorded in Table 1.

### 3.2. Simulation Model and Mesh Division

**Figure 7:**
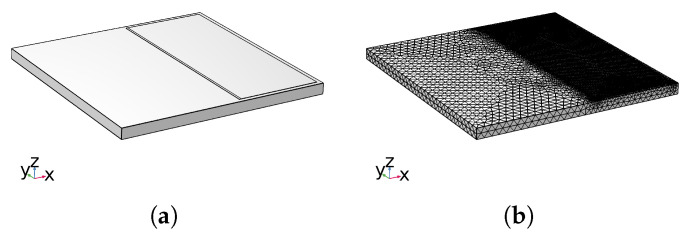
Simulation model and mesh division. (**a**) Simulation model, (**b**) mesh division.

In order to analyze the cracking factors of the atomizer, the relationship between temperature distribution and time was studied based on the model shown in Figure 7a. The finite element analysis method was used to establish the surface heat transfer and thermal stress analysis model of the atomizer in the figure. The model mainly includes two parts: a piezoelectric substrate (128° YX LiNbO${}_{3}$) and a liquid film in the atomization zone on the substrate surface. Due to the fact that the interface between the liquid film and the substrate is most susceptible to stress changes, therefore, this article mainly analyzes the stress changes at the interface between the liquid film and the substrate, omitting the modeling of the interdigital electrode section and simplifying the model.

The size of the piezoelectric substrate is 10 mm × 10 mm × 0.5 mm, and the material properties used are shown in Table 2. The liquid film part is located on the right surface of the piezoelectric substrate, with a size of 4 mm × 9.6 mm × 0.01 mm, considering that the liquid film has a thickness of micrometers, it can be ignored during the heat transfer process. Therefore, the key physical field in the entire model of the atomizer is solid heat transfer.

The constructed atomizer model has heat sources, heat conduction, and surface thermal convection on its surface. The key sorting expressions are shown in the following formulas.

Thermal conduction:

(1) $$Q_{c}=kA\frac{T-T_{a}}{d}$$

Thermal convection:

(2) $$Q_{v}=hA\left(T-T_{a}\right)$$

In the above equations, *k* represents thermal conductivity, *A* represents the heat transfer area, *T* represents the device temperature, $T_{a}$ represents the device temperature, *d* represents the thickness of the model, and *h* represents the convective heat transfer coefficient. After determining the temperature distribution of the atomizer, the stress and strain distribution can be calculated based on the material’s thermal expansion coefficient.

(3) $$\sigma =E(\varepsilon -\varepsilon_{T})$$

(4) ε=αΔT

In the equation, σ represents the stress of the substrate material, *E* represents Young’s modulus matrix, ε represents the strain, $\varepsilon_{T}$ represents the strain, α represents the thermal expansion coefficient, and ΔT represents the temperature difference.

In terms of mechanical constraints, the atomizer is bonded to the surface of a metal aluminum plate using a thermally conductive adhesive, so it is set as a fixed boundary. In terms of the heating process, in order to verify the correctness of the model, the fitting curve of the highest measured temperature was used as the input function of the heat source temperature. The temperature distribution on the atomizer surface was calculated through the model and compared with the measured results. In order to accurately reproduce the heating process of the atomizer, a heating zone corresponding to the measured high temperature area was set on the left side of the liquid film of the model, and its surface temperature was set as the segmented fitting value of the measured temperature. The remaining surface areas were set as natural convection on the upper surface, and the surrounding areas of the model were also set as natural convection on the sidewalls. The bottom of the model is set to conduct heat, and the thermal conductivity of the thermal conductive adhesive used at the bottom is about 5 W/(m${}^{2}\cdot$ k), while the thermal conductivity of the aluminum plate is 237 W/(m${}^{2}\cdot$ k). The actual thermal conductivity is related to the amount of thermal conductive adhesive used. When the simulation calculation is set to 10 W/(m${}^{2}\cdot$ k), the results obtained are similar to the actual experimental results.

Finally, the model was meshed (Figure 7b) using an adaptive meshing method, with the mesh unit size set to 0.015 mm in the liquid film boundary area. Through simulation testing and comparison, it was found that using a coarser mesh division (minimum unit 0.04 mm) can lead to deviation in the simulation results. The simulation results obtained using a 0.015 mm mesh are consistent with the results obtained using a more detailed mesh division method (minimum unit 0.002 mm). However, the size of a 0.002 mm mesh can increase the computational load and time of the model. Therefore, using a model with a unit size of 0.015 mm can quickly and accurately calculate the surface temperature and stress distribution of the atomizer for simulation.

### 3.3. Calculation Analysis

**Figure 8:**
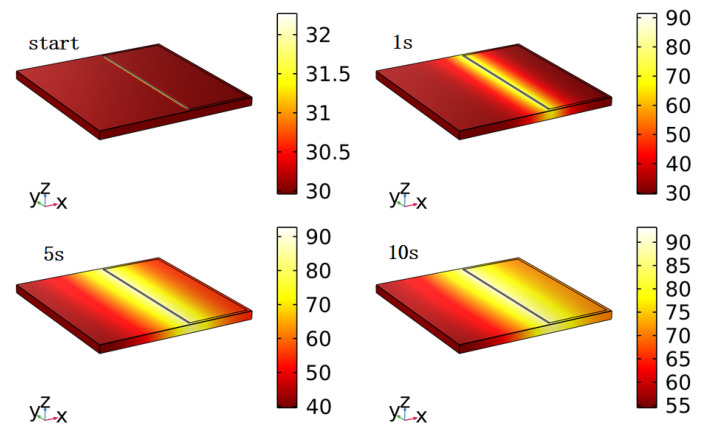
Simulation of temperature distribution.

During the simulation process, transient mode was used to calculate the surface temperature distribution of the atomizer at different times. Figure 8 shows the surface temperature distribution of the atomizer at system startup, 1 s, 5 s, and 10 s. From the figure, it can be seen that the surface temperature of the substrate gradually increases with time, and the surface temperature distribution gradually becomes uniform, but the temperature is highest at the boundary between the liquid film and the substrate. In the model, the highest temperature on the left side of the liquid film is set as the highest temperature value measured experimentally, while the temperature distribution on the surface of the model is obtained through simulation calculations. From Figure 8, it can be seen that after 1 s of operation, the temperature distribution in the right liquid film area is roughly between 90 °C and 30 °C. After 5 s, it increases to a range between 90 °C and 50 °C, and after 10 s, it further increases to a range between 90 °C and 65 °C. Compared with the measurement results of the infrared thermographic camera, it can be found that the calculated surface temperature distribution results of the model are basically consistent with the actual measurement results shown in Figure 4. According to Equations (3) and (4), the stress distribution can be determined by the material parameters and the temperature distribution. Therefore, it can be concluded that the stress results calculated using this model are consistent with the actual situation.

**Figure 9:**
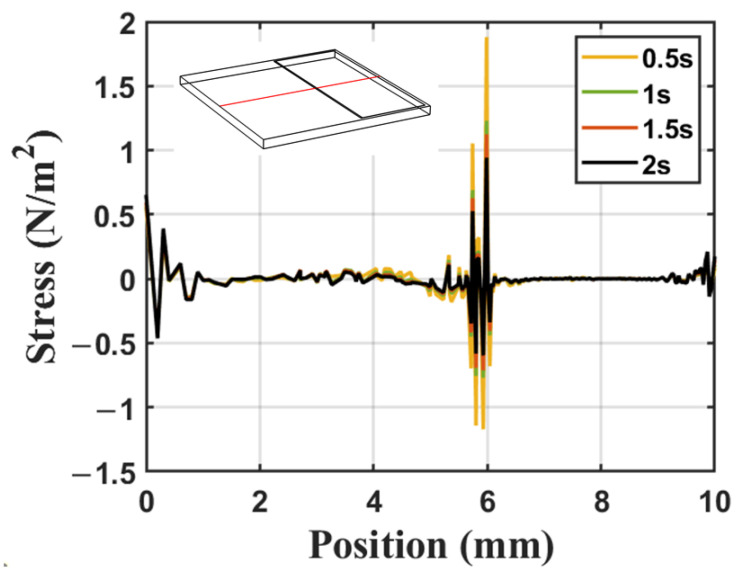
Stress distribution of the central line (red line in the model) in the surface.

In order to accurately analyze the surface stress distribution, the thermal stress on the sound propagation path (red line in the model in the upper left corner of Figure 9) is selected to represent the surface stress distribution of the atomizer. Taking the calculation results of water atomization at a power of 7 W as an example, Figure 9 shows the curve of the thermal stress distribution on the surface of the device over time. It can be seen from the graph that the Z-component of the stress tensor changes sharply near the boundary area between the liquid film and the substrate. Some particles are subjected to strong positive stress, while neighboring particles are subjected to strong negative stress, which can cause opposite pulling effects on the substrate and may induce cracks on the device surface.

The reason for the above results is that the temperature distribution on the surface of the substrate is uneven, and the expansion of the substrate at the heated part of the interface is hindered, which will result in negative stress, while adjacent particles in the area will be affected by positive stress due to the expansion of adjacent heated particles. By comparing the data at four different times from the graph, it can be observed that as the liquid film heats up and heat diffusion occurs throughout the atomizer, the stress in this region initially increases and then decreases, with maximum stress occurring around 0.4 s. This demonstrates that as the temperature gradually becomes uniform, the thermal stress on the substrate has also decreased.

**Figure 10:**
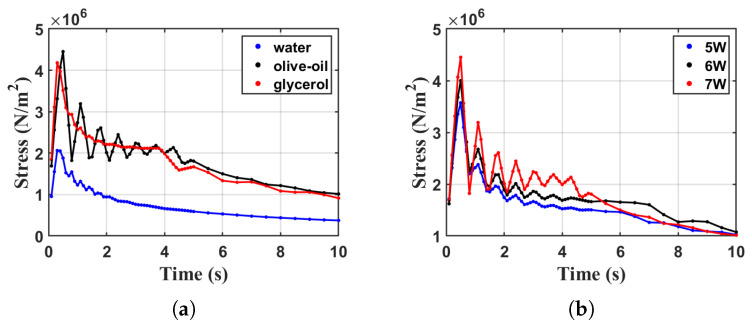
Stress peak vs. time variation. (**a**) Different liquid at 7 W Power, (**b**) olive oil at different power levels.

Figure 10 shows the calculation results of stress changes over time under several different conditions. It can be seen from the figure that the maximum stress value under all conditions will appear around 0.4 s, indicating that to reduce thermal stress on the atomizer’s surface, it is essential to pay special attention to the atomizer’s initial operational phase. Figure 10a depicts the simulation results of liquid stress under a 7-watt power drive condition. From the figure, it can be observed that water exhibits the smallest maximum stress among the three liquids, measuring approximately $2\times 10^{6}$ N/m${}^{2}$. This is attributed to the low viscosity and low boiling point of water, resulting in a slower temperature rise and a lower maximum temperature reached. Olive oil and glycerin, with their higher viscosity and higher boiling point, lead to higher surface temperatures of the device, causing greater stress, ranging from approximately $4\times 10^{6}$ N/m${}^{2}$ to $4.5\times 10^{6}$ N/m${}^{2}$. Figure 10b illustrates the stress calculation results for olive oil under three different power drive conditions. It can be observed from the figure that reducing power results in a lower stress peak. Under the 7 W condition, the stress peak for olive oil approaches approximately $4.5\times 10^{6}$ N/m${}^{2}$, while under the 5 W condition, it is approximately $3.5\times 10^{6}$ N/m${}^{2}$.

Based on the comparative analysis of calculation results and fitting curves, the primary cause of the stress peaks is attributed to an excessively rapid initial temperature rise. The initial temperature rise rates for the aforementioned three liquids can commonly reach 150 °C/s. Due to water’s lower boiling point, further temperature elevation is constrained, resulting in minimal stress. However, the other two liquids with higher boiling points maintain a relatively rapid temperature rise until reaching 140 °C as indicated by test data. Contrast the higher stress observed in olive oil and glycerol, although glycerol possesses greater viscosity and a faster rate of temperature rise, its thermal stress does not significantly differ from that of olive oil. The main reason is that both liquids can rapidly rise to 140 °C. Afterward, under the combined effect of accelerated heat exchange and reduced liquid viscosity, the heating rate begins to slow down, the temperature uniformity of the atomizer surface improves, and the thermal stress gradually decreases.

### 3.4. Optimization of Thermal Stress Resulting from Power Input

From the results and analysis presented above, it is evident that the thermal stress in the atomizer is primarily concentrated in the edge region of the wicking material. Moreover, during the initial phase of atomization, the stress reaches its maximum level. Therefore, it is recommended to appropriately lower the input power and the rate of temperature rise during the initial phase to prevent excessive thermal stress. In order to determine the effect of heating rate on stress, this article used seven different heating curves for simulation calculations. The following is a detailed introduction to the setting method of the heating curve.

**Figure 11:**
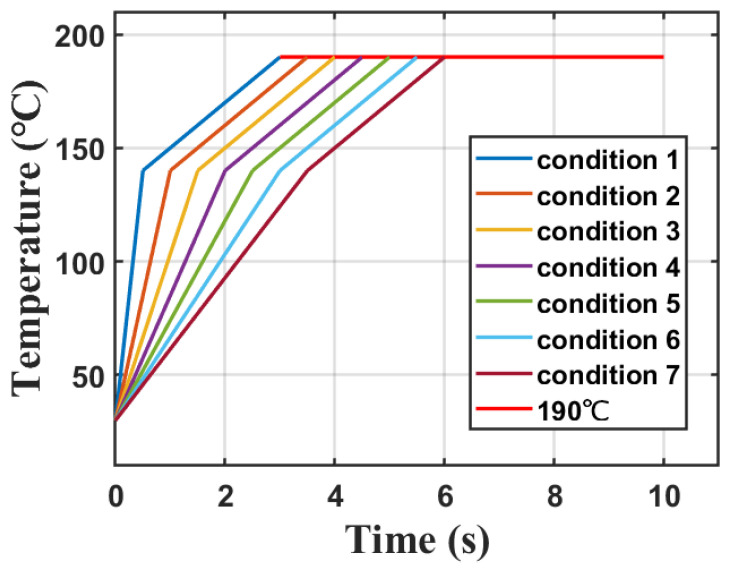
Heating curves under seven different conditions.

Observing the fitting curves (Figure 6) and stress peak graphs (Figure 10), it can be noted that for liquid with a higher boiling point and viscosity, after reaching the highest temperature, they will maintain around 190 °C. Therefore, the maximum value of the third temperature curve is set at a constant value of 190 °C. After reaching 140 °C approximately, the rate of temperature rise slows down. The fitting curve (Table 1) shows that the rate of temperature rise in this segment is approximately 15 °C/s. Considering that a faster temperature rise results in higher stress, the rate of temperature rise in the second stage is set at 20 °C/s, with a temperature range of 140 °C to 190 °C in order to ensure that the actual stress is lower than the simulated stress. Temperature control should primarily focus on the first stage of heating to approximately 140 °C, assuming this stage is a linear temperature rise. By setting different rates of temperature rising, the maximum stress can be calculated using the above model. As shown in Figure 11, seven different heating curves are calculated, assuming the first stage of heating lasts from 0.5 s to 3.5 s individually, with intervals of 0.5 s. The key parameters summarized are shown in Table 3.

**Figure 12:**
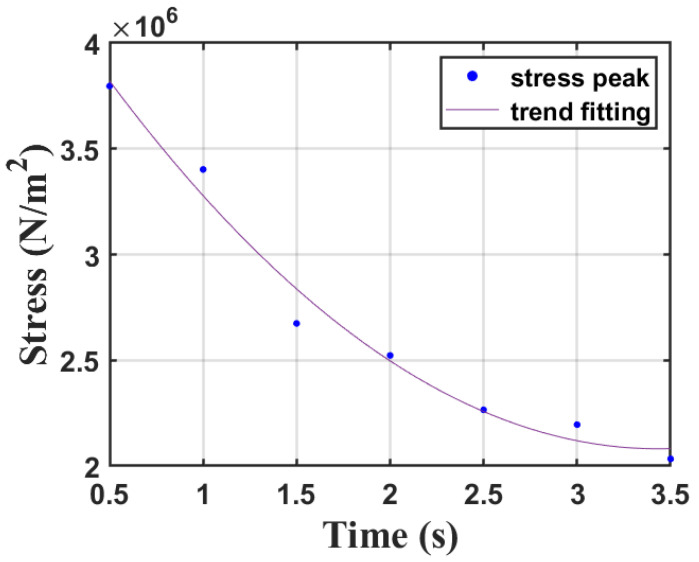
Thermal stress simulation results under 7 conditions.

By inputting the aforementioned heating curves into the model and calculating the maximum stress under the corresponding conditions, the results are plotted in Figure 12. It can be observed from the graph that as the duration of heating increases and the heating rate slows down, the thermal stress on the atomizer’s surface gradually decreases. Considering that substrate breakage did not occur in multiple water atomization experiments, the maximum stress reached in the water atomization, which is about $2\times 10^{6}$ N/m${}^{2}$, serves as the target for stress control.

Comparing the curve in Figure 12 with Table 3, it can be seen that if the temperature rises linearly from 30 °C to 140 °C, the input power needs to be controlled within the first 3.5 s (condition 7) to ensure that the thermal stress on the substrate surface is about $2\times 10^{6}$ N/m${}^{2}$. In consideration of the actual heating conditions, it is important to note that as the temperature increases, it leads to an acceleration in thermal convection. Consequently, the heating process of the atomizer generally does not follow a linear pattern. Therefore, obtaining actual stress values in experimental settings still requires a combination of surface temperature measurements and model calculations.

### 3.5. Verification Experiment

**Figure 13:**
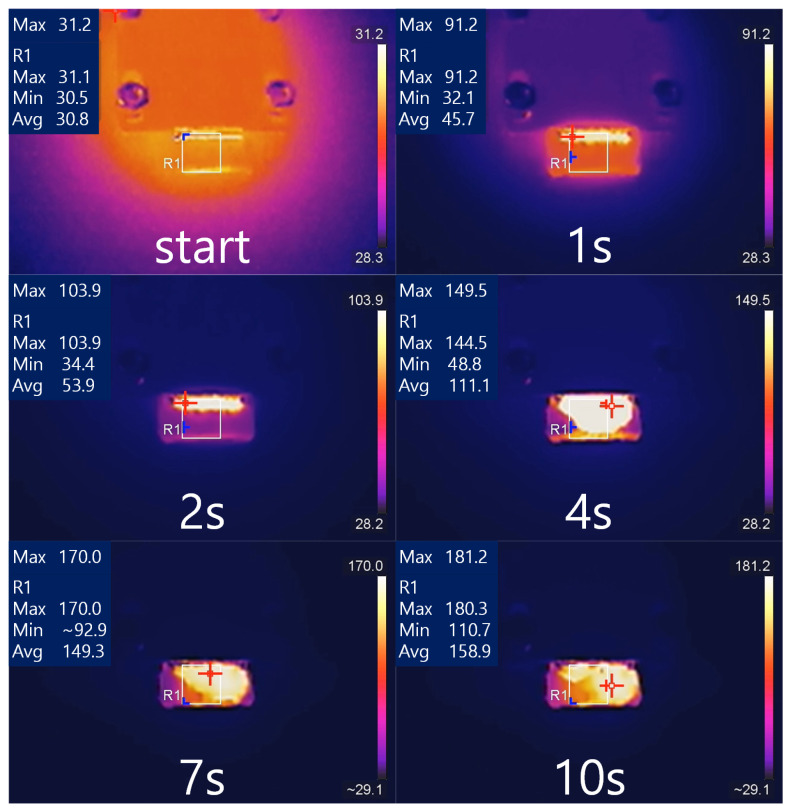
Temperature distribution under control: the red symbol (the highest point); the blue symbol (the lowest point).

According to previous experimental tests, it is known that in the currently used atomizer structure, several olive oil atomization experiments driven by 7 W power can cause substrate rupture. Therefore, based on the simulation results, this article designed a simple power control method and retested the atomizer operation under 7 W conditions. The power control method used in the experiment is to apply a 3 W power drive in the first 2 s, and then apply a 7 W power drive. The atomized liquid is olive oil. Figure 13 shows the temperature measurement results of the infrared thermographic camera of the atomizer.

From the temperature measurement results of the first and second seconds, it can be seen that if the driving power is controlled to 3 W, the maximum temperature on the surface of the atomizer will reach about 91 °C after 1 s and about 104 °C after 2 s. The initial temperature at the beginning of the experiment is about 30 °C, so the heating rate in the first second is about 60 °C/s, and the heating rate in the second second is about 10 °C/s. The temperature measurement result in the 4th second is 144 °C, with a heating rate of 20 °C/s for 2 to 4 s. It reaches 170 °C in the 10th second and gradually stabilizes, with a heating rate of 6 °C/s for 4 to 10 s. The parameters of the segmented temperature fitting curve are shown in Table 4. After incorporating the aforementioned heating parameters into the model, the calculated peak stress occurs at 1.1 s with a magnitude of approximately $1.85\times 10^{6}$ N/m${}^{2}$, which is less than $2\times 10^{6}$ N/m${}^{2}$, aligning with the expected control standards.

The method described in this paper was applied in multiple repeated experiments. Considering that the atomizer reaches its maximum temperature and stabilizes its operation in around 10 s, each operating cycle was set as follows: 3 W operation for 2 s, followed by 7 W operation for 18 s, finally cooling for 2 min and 40 s, totaling 3 min. This ensured that each working cycle could thoroughly test the atomizer’s heating phase, stable operating phase, and cooling phase.

These tests were carried out using an automated control program, and as of now, this study has completed over 5 h (more than 100 cycles) of testing without any atomizer ruptures. This significantly exceeds the previous number of rupture experiments conducted on atomizers, demonstrating the effectiveness of the method described in this paper in mitigating excessive thermal stress during the heating process.

## 4. Conclusions

This study investigated the influence of the rate of temperature rise on the thermal stress of the surface acoustic wave atomizer and proposed a control strategy through simulation experiments. By analyzing experimental data, we observed that during the temperature rise from room temperature to 140 °C, the thermal stress generated inside the material may have an adverse effect on the atomizer’s performance and stability. An excessive temperature rise rate can lead to a sharp change in substrate temperature distribution, resulting in concentrated thermal stress. Through numerical simulation and experimental analysis, this study verified the relationship between the rate of temperature rise and the increase in thermal stress, revealing the physical mechanism of this relationship.

The results of this study show that during the temperature rising stage from room temperature to 140 °C, rapid temperature rise can easily generate excessively higher thermal stress. By controlling the rate of temperature rise during this stage, it is possible to effectively reduce thermal stress and minimize the risk of atomizer breakage. Specifically, by controlling the drive power within the first 4 s after the initial operation to ensure that the rate of temperature rise does not exceed 31.4 °C/s, thermal stress can be maintained below $2\times 10^{6}$ N/m${}^{2}$, which is close to the thermal stress generated during water atomization at 7 W power, and then significantly extends the lifespan of the atomizer. Finally, this article conducted repeated tests using a simple temperature control method, and the results of multiple tests proved that this method indeed extended the service life of the atomizer. This conclusion provides valuable guidance for the design and operation of SAW atomization systems. Nevertheless, many factors may affect the formation of thermal stress, such as material properties and environmental conditions. Therefore, further research and optimization are needed for specific designs when this strategy is implemented.

## Figures and Tables

**Table 1:** Parameters table for curve fitting.

Liquids	Power Levels	Stage 1	Stage 2	Stage 3
Olive Oil	5 W	y = 188.71x + 46.29	y = 7.44x + 127.97	y = 1.69x + 163.18
Olive Oil	6 W	y = 221.08x + 40.11	y = 9.02x + 134.51	y = 0.78x + 189.46
Olive Oil	7 W	y = 242.88x + 38.99	y = 14.25x + 134.27	y = −0.04x + 194.51
Water	7 W	y = 159.32x + 32.27	y = 4.65x + 85.62	y = 0.11x + 92.09
Glycerol	7 W	y = 330.52x + 31.61	y = 14.49x + 136.09	y = −0.23x + 189.78

**Table 2:** The 128° YX LiNbO${}_{3}$ simulation material parameters.

Parameter	Value	Unit
Density	4659	kg/m${}^{3}$
Thermal conductivity	5.6	W/(m · k)
Constant pressure heat capacity	800	J/(kg · K)
Young’s modulus	$130\times 10^{9}$	Pa
Poisson’s ratio	0.33	
Coefficient of thermal expansion	$5.5\times 10^{-6}$	1/K

**Table 3:** Key parameters for seven heating conditions used for simulation.

	Stage 1	Stage 1	Stage 2	Stage 2	Stage 3
	Time	Heating Rate	Time	Heating Rate	Temperature
Condition 1	0.5 s	220 °C/s	2.5 s	20 °C/s	190 °C
Condition 2	1 s	110 °C/s	2.5 s	20 °C/s	190 °C
Condition 3	1.5 s	73.3 °C/s	2.5 s	20 °C/s	190 °C
Condition 4	2 s	55 °C/s	2.5 s	20 °C/s	190 °C
Condition 5	2.5 s	44 °C/s	2.5 s	20 °C/s	190 °C
Condition 6	3 s	36.7 °C/s	2.5 s	20 °C/s	190 °C
Condition 7	3.5 s	31.4 °C/s	2.5 s	20 °C/s	190 °C

**Table 4:** Parameters table for segmented temperature fitting curve.

	Duration	Curve
Stage 1	1 s	y = 60x + 30
Stage 2	1 s	y = 10x + 80
Stage 3	2 s	y = 20x + 60
Stage 4	6 s	y = 6x + 116

## Data Availability

Not applicable.
